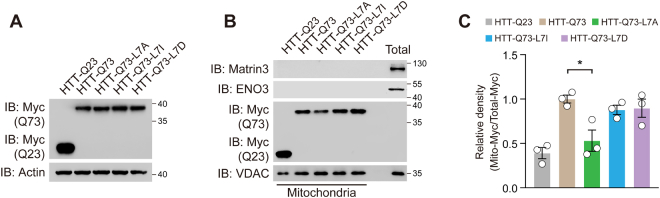# Leucine 7 is a key residue for mutant huntingtin–induced mitochondrial pathology and neurotoxicity in Huntington's disease

**DOI:** 10.1016/j.jbc.2025.108297

**Published:** 2025-02-11

**Authors:** Shengrong Zhang, Shengda Wang, Zeyue Yang, Yuanbo Li, Jinping Li, Xushen Chen, Hao Yao, Zhilong Zheng, Xing Guo

**Affiliations:** 1 Department of Neurobiology, Key Laboratory of Human Functional Genomics of Jiangsu Province, School of Basic Medical Sciences, Nanjing Medical University, Nanjing, Jiangsu, China; 2 Department of Anesthesiology, The Second Affiliated Hospital of Nanjing Medical University, Nanjing, Jiangsu, China; 3 State Key Laboratory of Reproductive Medicine and Offspring Health, Nanjing Medical University, Nanjing, Jiangsu, China; 4 The Second Affiliated Hospital of Nanjing Medical University, Nanjing, Jiangsu, China; 5 Co-innovation Center of Neuroregeneration, Nantong University, Jiangsu, China

**Keywords:** Huntington's disease, mutant huntingtin, N17 domain, mitochondria, neurodegeneration

## Abstract

Huntington's disease (HD) is a neurodegenerative disorder caused by the abnormal expansion of CAG repeats in exon 1 of the *HTT* gene. Mutant huntingtin (mHTT) associates with mitochondria, resulting in mitochondrial dysfunction and neuronal cell death. However, the underlying molecular mechanisms remain unknown. In this study, we investigate the role of N-terminal first 17 amino acids (N17) of mHTT in regulating its mitochondrial localization. Specifically, we demonstrate that the mutation at leucine 7 of N17 domain suppresses the association of mHTT with mitochondria. Blocking mitochondrial localization of HTT exon 1 with 73 glutamine repeats (HTT-Q73) strongly ameliorates polyglutamine-induced reduction of mitochondrial membrane potential, increase of reactive oxygen species production, and decrease in NAD^+^/NADH ratio. We observe that HTT-Q73-mediated abnormal mitochondrial morphology, mitochondrial DNA deletion, and cell death are abolished by HTT-Q73-L7A mutation. Finally, overexpression of HTT-Q73-L7A do not cause neurodegeneration and motor dysfunction *in vivo*. These findings highlight the pivotal role of the L7 residue which contributes to mHTT-caused HD pathology. Targeting the L7 residue of N17 domain may be a novel therapeutic strategy to alleviate mitochondrial dysfunction and neurodegeneration in HD.

Huntington's disease (HD) is a dominantly inherited neurodegenerative disorder characterized by involuntary choreiform movements and progressive cognitive and psychiatric decline (1, 2). HD is caused by an abnormal expansion of CAG repeats in exon 1 of the *HTT* gene on chromosome 4, which leads to the production of mutant huntingtin (mHTT) protein containing expanded polyglutamine (polyQ) tracts (3, 4). CAG repeats above 36 may cause HD, while repeats over 40 result in full penetrance (5, 6). Despite HTT being ubiquitously expressed, postmortem studies consistently identify the striatum and cerebral cortex as the most severely affected regions in HD (7, 8), though the mechanisms underlying this selective vulnerability remain unclear.

Defect in energy metabolism, particularly mitochondrial dysfunction, is a hallmark of HD in both patient and animal models, closely linked to the loss of striatal neurons (9, 10, 11). The mitochondrial translocation of mHTT is a primary driver of mitochondrial damage, leading to depolarization (12), fragmentation (13), mitochondrial DNA (mtDNA) instability (10), mitophagy impairment (14, 15), and other dysfunctions. However, the underlying molecular mechanism remains to be investigated. The translocase of inner mitochondrial membrane 23 is reported to interact with mHTT, resulting in mitochondrial import defect and subsequent neuronal cell death (16). Our previous work demonstrated that mitochondrial-localized mHTT recruits valosin-containing protein to mitochondria, thereby impairing mitophagy and contributing to neurodegeneration (14). Furthermore, mHTT has also been proved to interact with mitochondrial fission GTPase dynamin-related protein-1 (DRP1) and enhance its enzymatic activity to trigger mitochondrial fragmentation (17). The aforementioned evidence underscores the intricate interplay between mHTT and mitochondrial dysfunction, indicating that disrupting the interaction between mHTT and mitochondria may present novel therapeutic avenues for alleviating neurodegeneration in HD.

The N-terminal first 17 amino acids (N17) of HTT exon 1 (Httex1) forms an amphipathic alpha-helix that is critical for polyQ oligomerization and aggregation (18, 19, 20). Phosphorylation of serine 13 and serine 16 attenuates polyQ aggregation and provides neuroprotection in HD models (21, 22). Additionally, a study using BAC transgenic mice expressing mHTT lacking the N17 domain have demonstrated aggravated polyQ aggregation and accelerated neurodegeneration (23), underscoring the complex, context-dependent role of N17 domain in HD pathogenesis. Recent findings further reveal that free N17 peptides or their analogs can inhibit both polyQ aggregation and its interaction with lipids (24, 25), suggesting that N17 domain not only modulates aggregation but also affects other aspects of HD pathology. In addition to its role in aggregation, this domain also acts as a subcellular targeting signal that regulates nuclear trafficking of mHTT (23, 26). Considering that classical mitochondrial localization signals typically reside at the N terminus of proteins (27, 28), we hypothesize that N17 domain may play a critical role in mitochondrial translocation of mHTT. Targeting this sequence could represent a novel approach to disrupt the interaction between mHTT and mitochondria, potentially mitigating neurodegeneration in HD.

By substituting the leucine residue at position 7 (L7) within the N17 domain with alanine, a minimally disruptive amino acid, we observed a marked reduction in mHTT's mitochondrial localization. This substitution highlights the pivotal role of L7 in mediating mHTT translocation, leveraging alanine's minimal impact on polarity, protein folding, and stability. Furthermore, using both *in vivo* and *in vitro* models, we demonstrate that the L7A mutation within N17 domain mitigates polyQ-induced mitochondrial dysfunction. Specifically, the mutation restores mitochondrial membrane potential (MMP), reduces mitochondrial reactive oxygen species (mtROS) production, and normalizes the NAD^+^/NADH ratio. Additionally, L7A mutation prevents polyQ-driven abnormal mitochondrial morphology, mtDNA depletion, and cell death, ultimately rescuing neurodegeneration and improving locomotor activity.

## Results

### Mutation at leucine 7 of N17 domain suppresses the mitochondrial association of HTT exon 1 with 73 glutamine repeats

**Figure 1:**
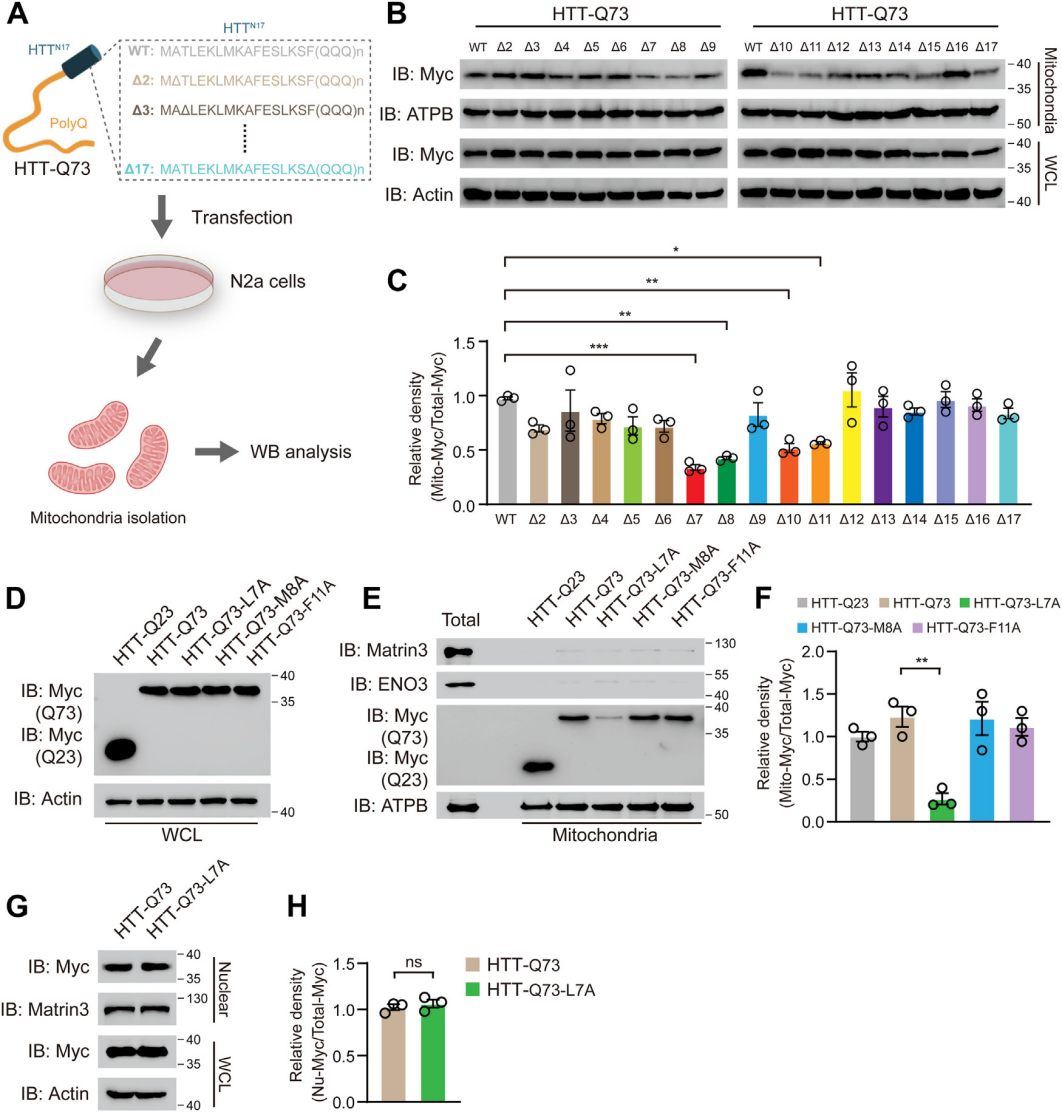
**Mutation at leucine 7 of N17 domain suppresses HTT-Q73 association with mitochondria**. *A*, schematic representation of detecting mitochondrial localization of HTT-Q73 mutants. *B* and *C*, Western blotting and quantitative analysis of Myc-tagged wildtype (WT) HTT-Q73 and N17 domain-truncated HTT-Q73 mutants in mitochondrial fractions of N2a cells at 72 h post-transfection. n = 3 independent experiments. ATPB: loading control of mitochondrial fractions; Actin: loading control of whole cell lysates (WCLs). One-way ANOVA was conducted, followed by Dunnett's multiple comparisons test (related to HTT-Q73-WT). *D*, Western blotting of the expression of HTT-Q73 mutants (WT, L7A, M8A, and F11A) and HTT-Q23 in WCL of N2a cells at 72 h post-transfection. *E* and *F*, Western blotting and quantitative analysis of the expression of HTT-Q73 mutants in mitochondrial fractions of N2a cells at 72 h post-transfection. n = 3 independent experiments. Matrin3: loading control of nuclear fractions; ENO3: loading control of cytosol fractions. One-way ANOVA was conducted, followed by Tukey's multiple comparison test. *G* and *H*, Western blotting and quantitative analysis of the expression level of Q73-L7A mutant in nuclear fractions and WCL of N2a cells at 72 h post-transfection. Unpaired *t* test was conducted. n = 3 independent experiments. ∗*p* < 0.05; ∗∗*p* < 0.01; ∗∗∗*p* < 0.001. HTT-Q73, HTT exon 1 with 73 glutamine repeats.

To test whether the N17 domain is required for mHTT association with mitochondria, we generated a series of N17 deletion mutants as shown in Fig. 1*A*. Mitochondrial fractions were isolated from N2A cells expressing HTT exon 1 with 73 glutamine repeats (HTT-Q73) or its deletion mutants (Fig. 1*A*). Western blotting analysis showed that as reported previously, HTT-Q73 strongly associated with mitochondria (Fig. 1*B*, lane 1). Interestingly, deletion mutants ΔL7, ΔM8, ΔA10, and ΔF11 markedly diminished the association of HTT-Q73 with mitochondria (Fig. 1, *B* and *C*). Next, we performed alanine-scanning mutagenesis to further confirm the amino acid residues which are crucial for the HTT-Q73 accumulation on the mitochondria. Replacement of the L7, M8, or F11 with Ala had no effect on the total protein levels of HTT-Q73 (Fig. 1*D*). The L7A mutation significantly decreased the protein levels of HTT-Q73 in mitochondrial fractions, whereas no changes were observed in cells expressing the other HTT-Q73 mutants (Fig. 1, *E* and *F*). Additionally, we replaced the L7 residue with isoleucine or aspartic acid in HTT-Q73, but these substitutions did not affect the mitochondrial association of HTT-Q73 (Fig. S1, *A*–*C*). Previous studies have revealed that the N17 domain is linked to nuclear export of HTT fragments (29, 30). To determine whether leucine 7 is required for HTT-Q73 nuclear localization, we isolated nuclear fraction from HTT-Q73- or HTT-Q73-L7A-expressing cells and found that HTT-Q73 nuclear proteins levels were not affected by L7A mutation (Fig. 1, *G* and *H*). Taken together, these findings indicate that leucine 7 in N17 domain is responsible for mHTT mitochondrial localization.

### HTT-Q73-L7A mutation abolishes HTT-Q73-induced mitochondrial dysfunction

**Figure 2:**
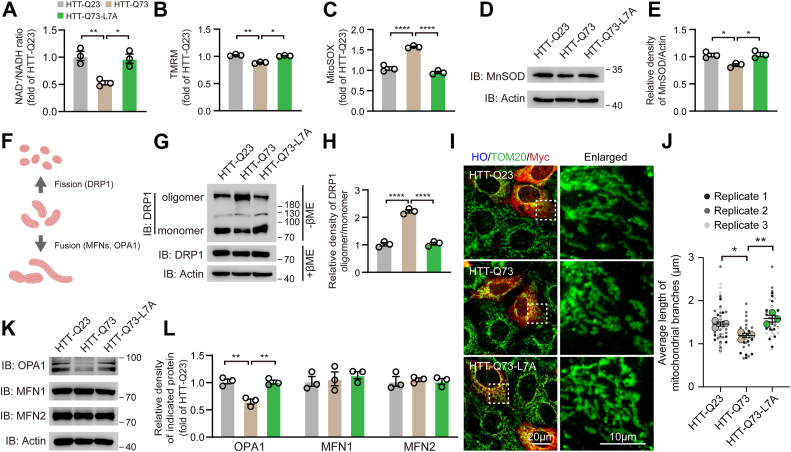
**HTT-Q73-L7A mutation abolishes HTT-Q73 induced mitochondrial dysfunction**. *A*–*C*, measurement of NAD^+^/NADH ratio, mitochondrial membrane potential (TMRM), mitochondrial reactive oxygen species (MitoSOX) in HTT-Q23-, HTT-Q73-, and HTT-Q73-L7A-transfected N2a cells at 72 h post-transfection. n = 3 independent experiments. *D* and *E*, Western blotting and quantitative analysis of the expression of MnSOD in HTT-Q23-, HTT-Q73-, and HTT-Q73-L7A-transfected N2a cells at 72h post transfection. n = 3 independent experiments. *F*, schematic representation of proteins involved in mitochondrial dynamics, including DRP1 (fission), and MFNs, OPA1 (fusion). *G* and *H*, Western blotting and quantitative analysis of DPR1 oligomerization in N2a cell lysate without βME at 72 h post transfection with HTT-Q23, HTT-Q73, and HTT-Q73-L7A. n = 3 independent experiments. *I* and *J*, Western blotting and quantitative analysis of the expression of OPA1, MFN1, and MFN2 in HTT-Q23-, HTT-Q73-, and HTT-Q73-L7A-transfected N2a cells at 72 h post-transfection. n = 3 independent experiments. *K* and *L*, representative images of mitochondrial morphology (TOM20 in *green channel*) and quantitative analysis of average length of mitochondrial branches in HTT-Q23-, HTT-Q73-, and HTT-Q73-L7A (Myc in *red channel*)-transfected SH-SY5Y cells at 72 h post-transfection. n > 45 cells from three independent experiments (replicate 1, replicate 2, and replicate 3). One-way ANOVA was conducted in the above results, followed by Tukey's multiple comparison test. ∗*p* < 0.05; ∗∗*p* < 0.01; ∗∗∗∗*p* < 0.0001. DRP1: dynamin-related protein-1; HTT-Q73, HTT exon 1 with 73 glutamine repeats; MFN, mitofusin; OPA1, optical atrophy 1.

Numerous studies have demonstrated that mHTT binds to mitochondria and induces mitochondrial dysfunction, including mitochondrial fragmentation, MMP loss, and mtROS production (31, 32). To explore whether the HTT-Q73-L7A mutation could prevent HTT-Q73-caused mitochondrial dysfunction, we transfected N2a cells with constructs expressing HTT-Q23, HTT-Q73, or HTT-Q73-L7A. We found that expression of HTT-Q73 led to a 49.5% reduction in NAD^+^/NADH ratio, a 13.9% reduction in MMP (measured by tetramethylrhodamine), and a 50.5% increase in mtROS, all of which were corrected by HTT-Q73-L7A mutation (Fig. 2, *A*–*C*). Manganese superoxide dismutase (MnSOD), a mitochondrial antioxidant enzyme, was downregulated by HTT-Q73 overexpressing. However, overexpression of HTT-Q73-L7A did not affect MnSOD levels compared with those cells expressing HTT-Q23, suggesting that HTT-Q73 elicited mtROS, at least in part, by reducing mtROS scavenging capacity (Fig. 2, *D* and *E*). We next asked whether HTT-Q73-L7A mutation could correct the abnormal mitochondrial dynamics in HD. As compared to HTT-Q73-transfected cells, the transfection of HTT-Q73-L7A effectively reversed the oligomerization of DRP1 and restored optical atrophy 1 (OPA1) expression, as well as increased the average length of mitochondrial branches (Fig. 2, *F*–*J*). However, we observed no changes in the expression levels of mitofusins (MFNs) after HTT-Q73 expressing (Fig. 2, *K* and *L*). Thus, these results suggest that the L7A mutation rescued mitochondrial dynamics which was impaired by polyQ repeat expansion.

### HTT-Q73-L7A mutation restores HTT-Q73-caused mtDNA deletion

**Figure 3:**
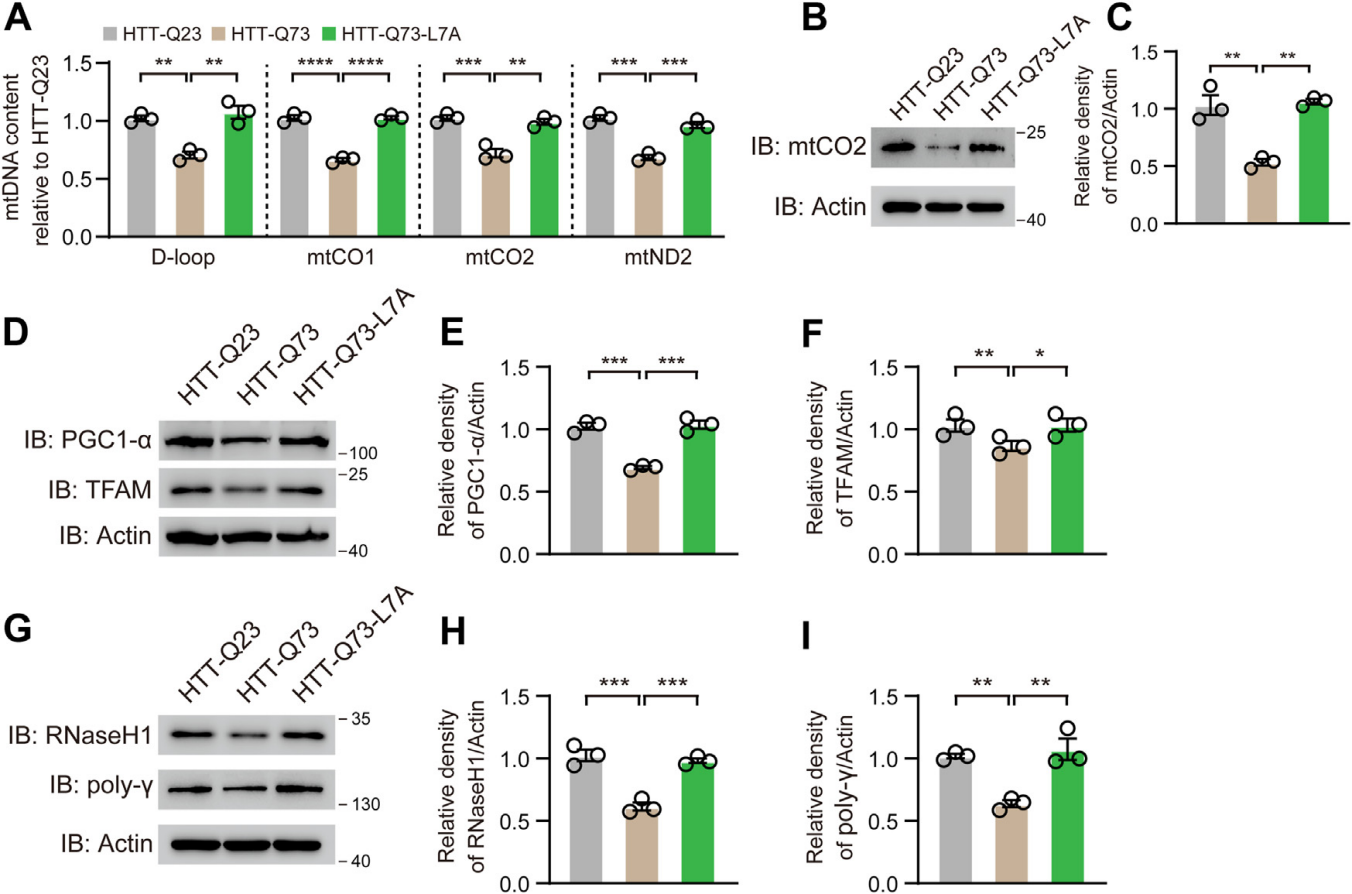
**HTT-Q73-L7A mutation restores HTT-Q73 caused mtDNA deletion**. *A*, RT-qPCR and quantitative analysis of the mtDNA contents (D-loop, mtCO1, mtCO2, and mtND2) in HTT-Q23-, HTT-Q73-, and HTT-Q73-L7A-transfected N2a cells at 72 h post-transfection. n = 3 independent experiments. *B* and *C*, Western blotting and quantitative analysis of mitochondria-encoding protein mtCO2 in HTT-Q23-, HTT-Q73-, and HTT-Q73-L7A-transfected N2a cells at 72 h post-transfection. *D*–*F*, Western blotting and quantitative analysis of the expression of PGC1-α and TFAM in HTT-Q23-, HTT-Q73-, and HTT-Q73-L7A-transfected N2a cells at 72 h post-transfection. n = 3 independent experiments. *G*–*I*, Western blotting and quantitative analysis of the expression of RNase H1 and poly-γ in HTT-Q23-, HTT-Q73-, and HTT-Q73-L7A-transfected N2a cells at 72 h post-transfection. n = 3 independent experiments. One-way ANOVA was conducted in the above results, followed by Tukey's multiple comparison test. ∗*p* < 0.05; ∗∗*p* < 0.01; ∗∗∗*p* < 0.001; ∗∗∗∗*p* < 0.0001. HTT-Q73, HTT exon 1 with 73 glutamine repeats; mtDNA, mitochondrial DNA; PGC-1α, peroxisome proliferator-activated receptor gamma coactivator 1-alpha; poly-γ, DNA polymerase γ; TFAM, mitochondrial transcription factor A.

Recent studies have highlighted the defects in mtDNA replication as critical factors in HD pathology (33, 34, 35). We next sought to address whether the HTT-Q73-L7A mutation improves HTT-Q73-induced mtDNA deletion. We used quantitative real-time PCR (qPCR) to compare mtDNA copy number and found that HTT-Q73-L7A mutation significantly restores the mtDNA content (Fig. 3*A*). Furthermore, we confirmed that mtDNA-encoded mitochondrially encoded cytochrome c oxidase II (mtCO2) is downregulated by HTT-Q73 expression, which was totally corrected by HTT-Q73-L7A mutation (Fig. 3, *B* and *C*). Peroxisome proliferator-activated receptor gamma coactivator 1-alpha (PGC1-α)/mitochondrial transcription factor A (TFAM) pathway drives the mtDNA replication and transcription, which is suppressed by mHTT (36). The quantification results showed that the expression levels of PGC1-α and TFAM in HTT-Q73-L7A mutant expressing cells were consistant with those in cells expressing control HTT-Q23 (Fig. 3, *D*–*F*). In addition, maintenance of mtDNA contents relies on a series of enzymes involved in DNA replication, such as ribonuclease H1 (RNaseH1) and DNA polymerase γ (poly-γ). Therefore, we hypothesized that these enzymes may be involved in HTT-Q73-L7A mutation-restored mtDNA content. To test this possibility, we evaluated the total protein levels and reveled that RNaseH1 and poly-γ levels in HTT-Q73-L7A-expressing cells are higher than that in HTT-Q73-expressing cells (Fig. 3, *G*–*I*). Thus, these data indicate that HTT-Q73-induced dysfunction of mtDNA replication machinery could be corrected by HTT-Q73-L7A mutation.

### HTT-Q73-L7A mutation reduces mHTT aggregation and HTT-Q73-induced neuronal cell death

**Figure 4:**
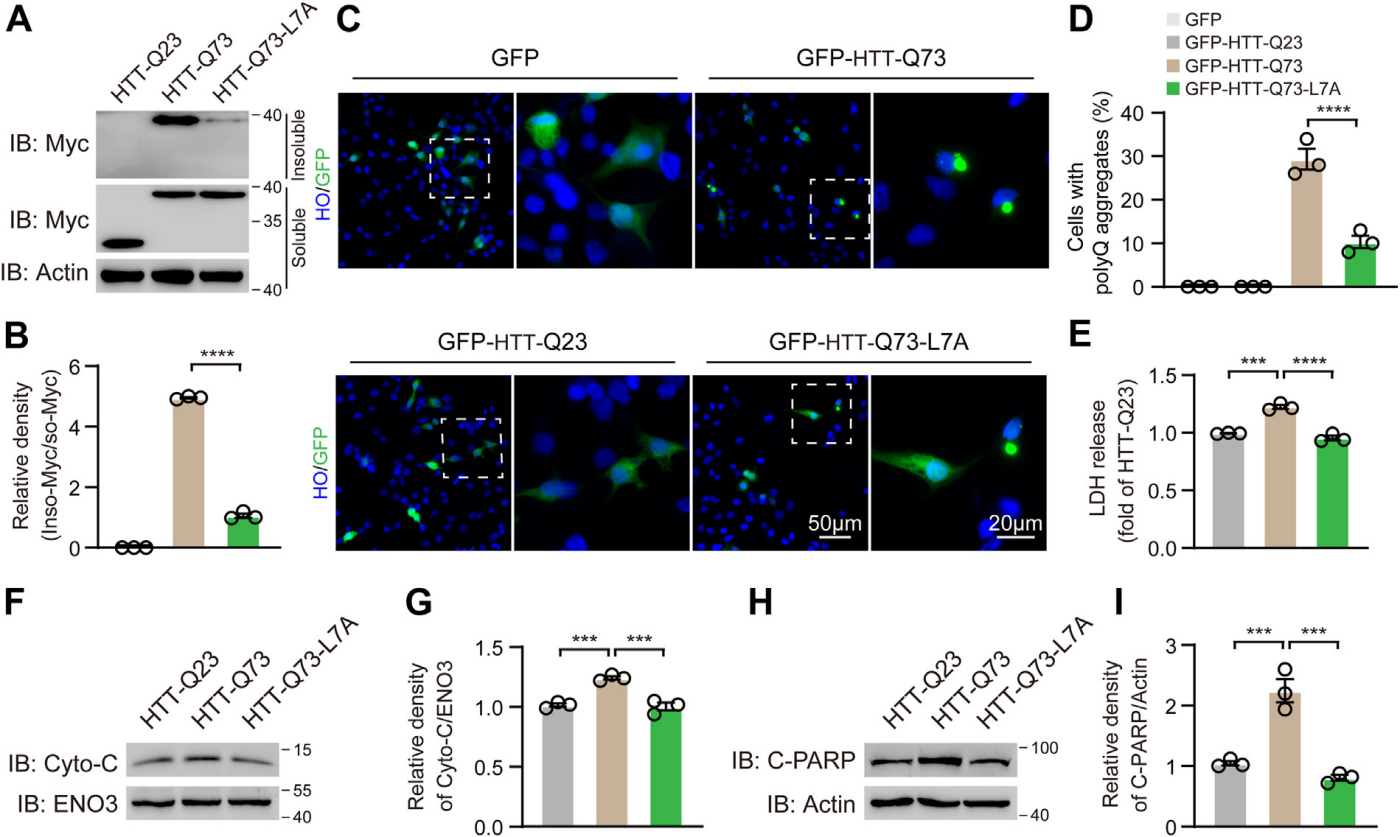
**HTT-Q73-L7A mutation reduces mHTT aggregation and HTT-Q73-induced neuronal cell death**. *A* and *B*, Western blotting and quantitative analysis of Myc-tagged HTT-Q23, HTT-Q73, and HTT-Q73-L7A in insoluble (inso-Myc) and soluble (so-Myc) fractions of HTT-Q23-, HTT-Q73-, and HTT-Q73-L7A-transfected N2a cells at 72 h post-transfection. n = 3 independent experiments. *C* and *D*, immunofluorescence and quantitative analysis the percentage of GFP-positive cells with polyQ aggregates (GFP-positive aggregates in *green channel*) in HTT-Q23-, HTT-Q73-, and HTT-Q73-L7A-transfected N2a cells at 72 h post-transfection. The proportion of polyQ aggregates present in GFP-positive cells within 10 random fields for each experiment was calculated. Nuclear was stained by Hoechst in *blue*. n = 3 independent experiments. *E*, measurement of LDH release in HTT-Q23-, HTT-Q73-, and HTT-Q73-L7A-transfected N2a cells at 72 h post-transfection. n = 3 independent experiments. *F*–*I*, Western blotting and quantitative analysis of the expression of Cytochrome C (Cyto-C) and cleaved PARP (C-PARP) in HTT-Q23-, HTT-Q73-, and HTT-Q73-L7A-transfected N2a cells at 72 h post-transfection. n = 3 independent experiments. One-way ANOVA was conducted in the above results, followed by Tukey's multiple comparison test. ∗∗∗*p* < 0.001; ∗∗∗∗*p* < 0.0001. GFP, green fluorescent protein; HTT-Q73, HTT exon 1 with 73 glutamine repeats; LDH, lactate dehydrogenase; mHTT, mutant huntingtin; PARP, poly ADP-ribose polymerase; polyQ, polyglutamine.

Since N17 domain is associated with mHTT aggregation accumulation (19, 26, 30), it would be interesting to explore the effect of the L7A mutation on mHTT aggregation. Overexpression of HTT-Q73 in N2a cells led to a significant accumulation of polyQ in the insoluble fractions. However, HTT-Q73-L7A mutation markedly suppressed polyQ aggregation (Fig. 4, *A* and *B*). To further visualize the impact of L7A mutation on polyQ aggregation, constructs of N-terminal green fluorescent protein (GFP)-fused HTT-Q23, HTT-Q73, and HTT-Q73-L7A were employed. Immunofluorescence analysis revealed that cells expressing GFP-Q73-L7A exhibited substantially lower polyQ aggregates compared to those cells expressing GFP-Q73 (Fig. 4, *C* and *D*). Additionally, we assessed cell viability and observed that the HTT-Q73-L7A mutation significantly mitigated HTT-Q73-induced cytotoxicity, which was evidenced by reduced lactate dehydrogenase (LDH) release, cytochrome c release, and poly ADP-ribose polymerase (PARP) cleavage (Fig. 4, *E*–*I*). These findings suggest that the HTT-Q73-L7A mutation not only diminishes polyQ aggregation but also confers protection against mHTT-mediated cell death.

### HTT-Q73-L7A mutation mitigates HTT-Q73-induced mitochondrial dysfunction and neurodegeneration *in vivo*

**Figure 5:**
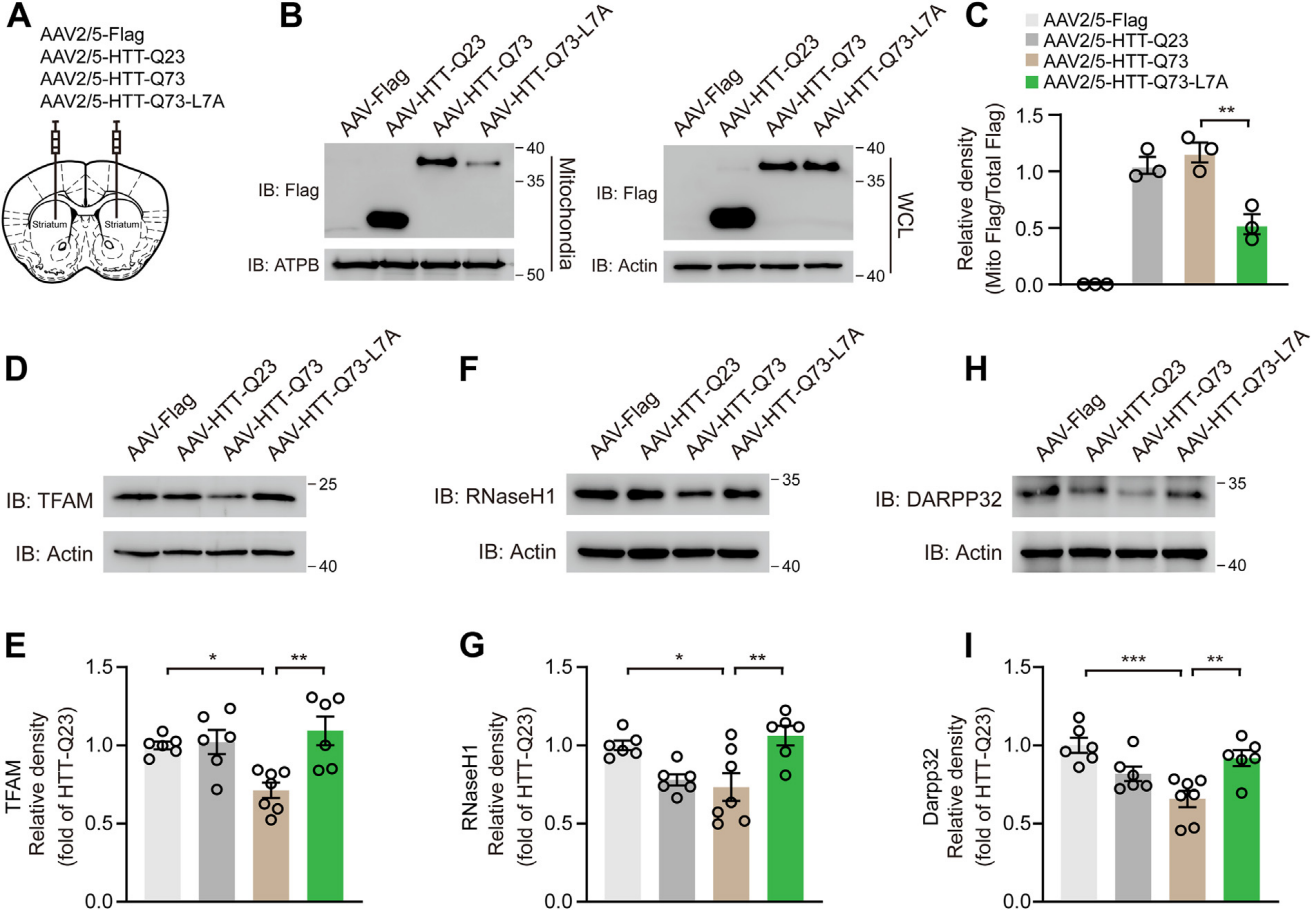
**HTT-Q73-L7A mutation mitigates HTT-Q73-induced mitochondrial dysfunction and neurodegeneration *in vivo***. *A*, schematic diagram of the injection of AAV2/5 in mouse striatum. *B* and *C*, Western blotting and quantitative analysis of the expression of flag-tagged HTT-Q23, HTT-Q73, and HTT-Q73-L7A in mouse striatum mitochondrial fractions and WCL at 6 weeks post AAV infection. n = 3 mice. *D*–*I*, Western blotting and quantitative analysis of the expression of DARPP32, TFAM, and RNaseH1 in mouse striatum at 6 weeks post AAV infection. n = 6 to 7 mice. One-way ANOVA was conducted in the above results, followed by Tukey's multiple comparison test. ∗*p* < 0.05; ∗∗*p* < 0.01; ∗∗∗*p* < 0.001. AAV, adeno-associated virus; HTT-Q73, HTT exon 1 with 73 glutamine repeats; RNaseH1, ribonuclease H1; TFAM, mitochondrial transcription factor A; WCL, whole cell lysates.

Next, we determined whether the HTT-Q73-L7A mutation would attenuate mHTT-associated mitochondrial dysfunction and neurodegeneration *in vivo*. Adeno-associated virus (AAV) was employed to virally overexpress HTT-Q23, HTT-Q73, or HTT-Q73-L7A in the striatum of 7-week-old WT mice (Fig. 5*A*). Two weeks after stereotaxic injection, we harvested the mitochondrial fractions from the striatum. Consistent with our *in vitro* findings, we observed a significant reduction in the abundance of HTT-Q73-L7A in mitochondrial fractions compared to HTT-Q73 (Fig. 5, *B* and *C*). Compared to the mice injected with AAV-HTT-Q73 control, immunoblot analysis indicated an obvious upregulation of mtDNA replication-related proteins TFAM and RNaseH1 in striatum lysates of AAV-HTT-Q73-L7A-injected mice (Fig. 5, *D*–*G*). Similarly, the protein levels of DARPP32, a marker of striatal medium spiny neurons, were significantly higher in AAV-HTT-Q73-L7A-injected mice, relative to those mice injected with AAV-HTT-Q73 (Fig. 5, *H* and *I*). These results suggest that the L7A mutation in N17 domain effectively reduces HTT-Q73-induced mitochondrial dysfunction and neurodegeneration *in vivo*.

### HTT-Q73-L7A mutation alleviates HTT-Q73-induced locomotor deficits *in vivo*

**Figure 6:**
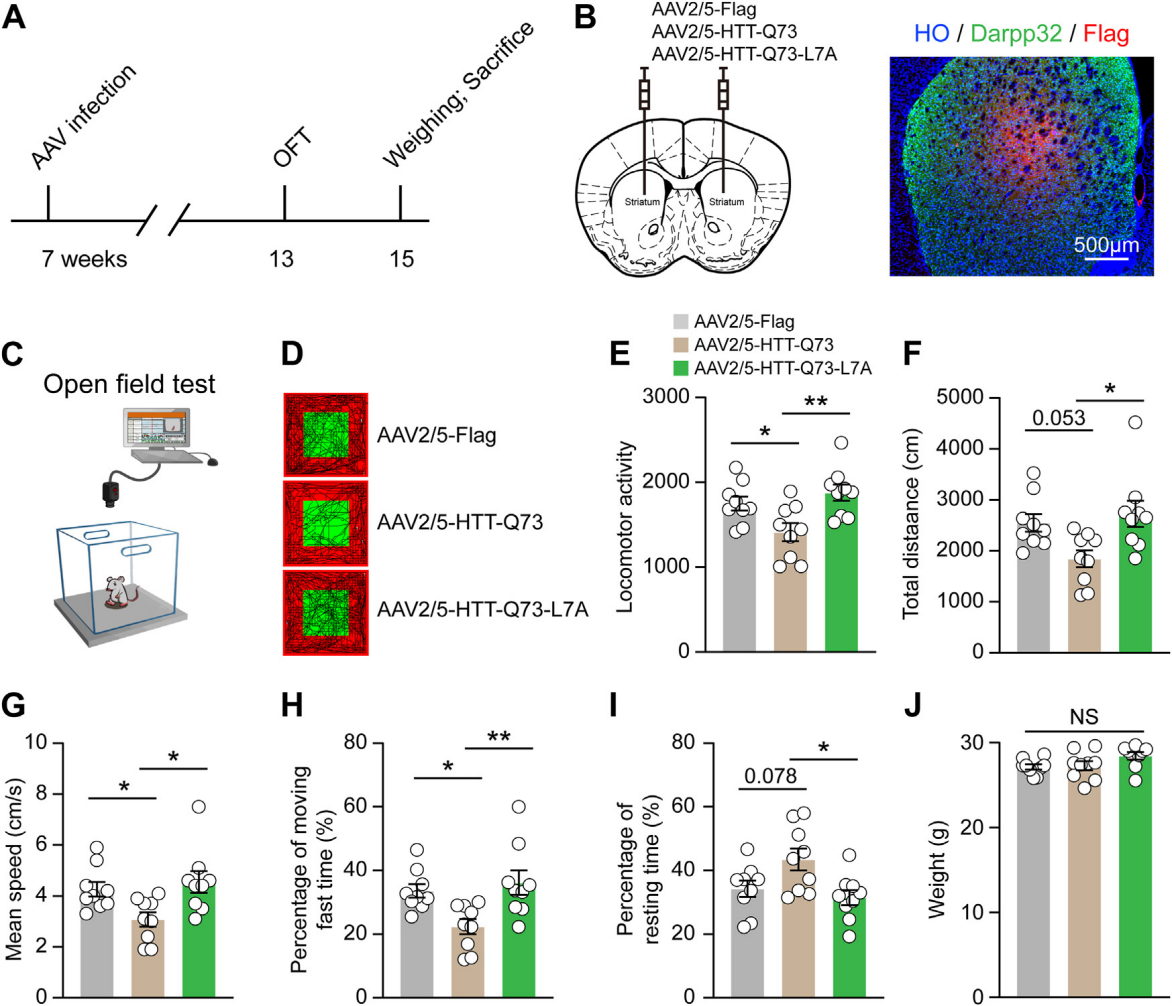
**HTT-Q73-L7A mutation alleviates HTT-Q73 induced locomotor deficits *in vivo***. *A*, schematic diagram of the open field test (OFT) and weighing on 7-week-old mice after AAV2/5-Flag, AAV2/5-Q23, AAV2/5-Q73, or AAV2/5-Q73-L7A infection for 6 weeks. *B*, representative image of DARPP32-positive cells with FLAG positivity in striatum of mice after AAV infection*. C* and *D*, a schematic diagram of OFT and the representative trajectory of AAV infected mice in OFT. *E*–*I*, OFT and quantitative analysis of the locomotor function in mice at 6 weeks post AAV infection. n = 9 mice. *J*, quantitatively analyze the weight changes of AAV infected mice. n = 9 mice. One-way ANOVA was conducted in the above results, followed by Tukey's multiple comparison test. NS, not significant; ∗*p* < 0.05; ∗∗*p* < 0.01. AAV, adeno-associated virus; HTT-Q73, HTT exon 1 with 73 glutamine repeats.

To evaluate the impact of the L7A mutation on HTT-Q73-induced locomotor function impairments, we overexpressed HTT-Q73 or HTT-Q73-L7A in the striatum of wildtype mice and assessed locomotor performance using the open field test (OFT) (Fig. 6, *A* and *C*). The expression of the constructs was confined to the DARPP32-positive striatal neurons. (Fig. 6*B*). Our results demonstrated that the L7A mutation significantly improved several locomotor performance metrics, including locomotor activity, total distance traveled, and mean speed, as compared to mice expressing HTT-Q73 (Fig. 6, *D*–*G*). Additionally, the percentages of both time spent moving fast and time spent resting were significantly increased in the HTT-Q73-L7A overexpression group (Fig. 6, *H* and *I*). Importantly, there was no significant body weight difference between the two groups, indicating that the observed effects were not due to differences in overall health or activity levels (Fig. 6*J*). These findings suggest that the L7A mutation in N17 domain effectively mitigates locomotor deficits induced by mHTT in mice.

## Discussion

Mitochondrial dysfunction is a central hallmark in the pathogenesis of HD, implicating multiple cellular processes including energy metabolism, oxidative stress, calcium homeostasis, and apoptosis (12, 33, 37). The mitochondrial translocation of mHTT has been identified as a pivotal event leading to this dysfunction. Despite the recognized role of the N17 domain in mHTT aggregation and its translocation to mitochondria, the precise molecular mechanisms underlying these processes remain poorly understood. This knowledge gap hinders the development of targeted therapies to mitigate mitochondrial dysfunction in HD.

Previous studies have suggested that phosphorylation at S13 and S16 and the competitive binding of N17 domain within mHTT can reduce polyQ aggregation (22, 24). However, their specific roles in the mitochondrial translocation of mHTT are not well delineated. Our findings offer new insights into the role of the L7 residue within N17 domain, demonstrating that the L7A mutation significantly decreases the mitochondrial localization of mHTT, thereby exerting protective effects on mitochondrial NAD^+^ metabolism and membrane potential, and reduces mtROS production. Nevertheless, the precise mechanisms by which the L7A mutation impairs mHTT translocation to mitochondria remain elusive. These effects may be mediated by changes in N17's structural conformation, its interactions with other proteins, or posttranslational modifications.

The dysregulation of mitochondrial dynamics, characterized by excessive fission and reduced fusion, is a common feature in neurodegenerative diseases, including HD (10, 38). Previous research has established a link between mitochondrial fragmentation in HD and the aberrant phosphorylation and oligomerization of DRP1 (33), alongside the downregulation of mitochondrial fusion protein OPA1 (39, 40). In this context, our study further explored the impact of the L7A mutation on these mitochondrial dynamics. We confirmed that the L7A mutation rescues the abnormal mitochondrial dynamics induced by HTT-Q73, as evidenced by reduced DRP1 oligomerization and restored expression level of OPA1. Furthermore, the L7A mutation also mitigated the polyQ-induced reduction in mtDNA replication and mtDNA copy number. These results not only reinforce the critical role of N17 domain in mHTT-mediated mitochondrial malfunction but also suggest a previously unrecognized involvement of the L7 residue in these processes.

Furthermore, we examined the neuroprotective effects of L7A mutation and observed that the mutation salvages medium spiny neurons in the striatum and improves locomotor dysfunction in HD models. Importantly, the mutation also significantly reduces polyQ aggregation and its accumulation in insoluble fractions. This reduction in aggregation may be attributed to the secondary effects of improved mitochondrial function or a direct involvement of the L7A mutation in modulating polyQ aggregation. These findings highlight the dual role of the L7 residue in both mitochondrial dynamics and polyQ aggregation, making it a compelling target for therapeutic intervention.

In summary, our findings reveal the crucial role of the L7 residue within the N17 domain of mHTT in mediating its mitochondrial translocation, a key contributor to mitochondrial dysfunction in HD. The L7A mutation effectively prevents mHTT from localizing to mitochondria, thereby rescuing mitochondrial dynamics, enhancing mtDNA replication, improving overall cellular health, and reducing neurodegeneration. Additionally, the mutation decreases polyQ aggregation, further underscoring the therapeutic potential of targeting the L7 residue. Future research should aim to elucidate the precise molecular mechanisms by which the L7A mutation disrupts mHTT-mitochondria interactions. This includes exploring the structural and biochemical alterations induced by the mutation and understanding its broader implications in the context of polyQ aggregation and HD progression. Such investigations could pave the way for novel therapeutic strategies that target specific residues within N17 domain, ultimately preventing mHTT-induced neurotoxicity and offering new hope for HD treatment.

## Experimental procedures

### Animals and stereotactic injection

All animals were maintained under a 12 h light/dark cycle. The experiments were carried out according to the standard protocols and were approved by the Animal Care and Use Committee at the Nanjing Medical University (IACUC2207055). Seven-week-old male littermates were selected for stereotactic brain injection as previous reported (33). Briefly, the mice were anesthetized and underwent surgery to expose their skulls. Three μL AAV (AAV2/5-hSyn-Flag, AAV2/5-hSyn-Q23-Flag, AAV2/5-hSyn-Q73-Flag, AAV2/5-hSyn-Q73-L7A-Flag) were injected in both sites of striatum (bregma: AP: 0.5 mm, ML: 2/-2 mm, DV: -3.5 mm) by using a stereotactic instrument (RWD, China). After injection, the wound was sutured, and the mice were placed on a prewarmed blanket until they awaked.

### Open field test

The mice underwent the OFT at 6 weeks postinfection with either AAV2/5-hSyn-Flag, AAV2/5-hSyn-HTT-Q73-Flag, or AAV2/5-hSyn-HTT-Q73-L7A-Flag. OFT was conducted in a square arena (50 cm × 50 cm, with 50 cm high walls) made of opaque Plexiglas. Mice were individually placed in the center of the arena and allowed to freely explore for 10 min. The arena was illuminated by a dim light (∼100 lux) to reduce potential stress. The movement of the mouse was tracked *via* video tracking software (ANY-maze, Stoelting). Parameters such as locomotor activity, total distance traveled, and mean speed were measured as indicators of locomotor function. The arena was thoroughly cleaned with 70% ethanol between trials to remove any olfactory cues.

### Cell culture

Mouse neuroblastoma cells (N2a) and human neuroblastoma cells (SH-SY5Y) were purchased from the American Type Culture Collection (ATCC). SH-SY5Y cells were maintained in Dulbecco's modified Eagle's medium (DMEM)-F12 (C11330500BT, Gibco). N2a cells were cultured in DMEM (SH30022.01, Cytiva). The above mediums were supplemented with 10% FBS (FSD500, ExCell Bio) and penicillin/streptomycin (100 mg/ml, E607011–0100, Sangon Biotech). Cells were grown in a 5% CO_2_ incubator at 37 °C.

### Constructs and transfection

Httex1-Q23-Myc (HTT-Q23) and Httex1-Q73-Myc (HTT-Q73) were obtained from the Coriell Institute for Medical Research. The pEGFP-N1 plasmid was maintained in our laboratory. Myc-tagged HTT-Q73 deletion mutants, including HTT-Q73Δ2, HTT-Q73Δ3, HTT-Q73Δ4, HTT-Q73Δ5, HTT-Q73Δ6, HTT-Q73Δ7, HTT-Q73Δ8, HTT-Q73Δ9, HTT-Q73Δ10, HTT-Q73Δ11, HTT-Q73Δ12, HTT-Q73Δ13, HTT-Q73Δ14, HTT-Q73Δ15, HTT-Q73Δ16, HTT-Q73Δ17, and the point mutants HTT-Q73-L7A, HTT-Q73-L7I, HTT-Q73-L7D, HTT-Q73-M8A, and HTT-Q73-F11A, were generated from HTT-Q73 by PCR-based site-directed mutagenesis. For transfection, plasmids were first mixed with serum-free DMEM. Transfection reagent (WIS 1600, GenEscort I) was then added at a ratio of 1:3 (plasmid to reagent volume), followed by gentle mixing. The mixture was incubated at room temperature (RT) for 15 min before adding to the cell culture medium. Cells were harvested 72 h post-transfection.

### Total protein extraction

For whole cell lysate of culture cells, cells were washed with an appropriate volume of phosphate buffered solution (PBS) 72 h post-transfection and then lysed on ice for 20 min using a total protein extraction buffer containing 50 mM Tris-HCl (pH 7.5), 150 mM NaCl, 1% Triton X-100, and protease inhibitors (1:100). For whole cell lysate of mouse striatum, indicated mice were sacrificed and dissected to obtain striatum tissue. The tissues were then homogenized and lysed on ice by above total protein extraction buffer. Protein lysate was centrifuged at 12,000 rpm for 10 min at 4 °C. The supernatant was collected and used as the total protein extract.

### Measurement of cellular viability and cytotoxicity

To evaluate cellular viability, we employed a NAD^+^/NADH detection kit for quantitative analysis. Briefly, cultured cells were washed with cold PBS for three times and then lysed to obtain cell lysate. The cell lysate was diluted to appropriate concentration, and the contents of NAD^+^ and NADH were measured according to the instructions (C0018M, Beyotime, Shanghai). To evaluate cytotoxicity, we used a LDH release assay kit to quantitatively analyze the extracellular release of LDH. Briefly, the culture cell supernatants were collected after treatment, and the contents of LDH were measured according to the instructions (C0017, Beyotime). The absorbance of the two measurements was measured by enzyme linked immunosorbent assay analyzer at 450 nm and 490 nm wavelengths, respectively.

### Isolation of mitochondria-enriched fractions

Mitochondria-enriched fractions were obtained as previously described (41). Briefly, cells or mouse striatum tissues were lysed with mitochondrial isolation buffer (210 mM mannitol, 70 mM sucrose, 1 mM EDTA, 10 mM Hepes [pH 7.5], and protease inhibitor) on ice for 10 min. The lysates were disrupted 20 times by repeated aspiration through a 25-gauge needle. The homogenates were then centrifuged at 700*g* for 5 min to remove the plasma membrane fraction. The supernatant was centrifuged once again at 700*g* for 5 min, followed by a centrifugation at 6000*g* for 10 min to isolate mitochondria-enriched fraction. The pellet was then washed with mitochondrial isolation buffer and centrifuged at 6000*g* for 10 min, subsequently be lysed in total lysis buffer for downstream analysis.

### Isolation of nuclear-enriched fractions

Forty-eight hours post-transfection, cells were harvested and washed with an appropriate volume of PBS. Subsequently, 1 ml of cytoplasmic lysis buffer (20 mM Hepes [pH 7.0], 10 mM KCl, 2 mM MgCl₂, 0.5% NP-40, and 10 mM NaF) was added, and the cells were lysed on ice for 20 min. The lysate was transferred to a 1.5 ml microcentrifuge tube using a cell scraper and sheared by passing through a 1 ml syringe 20 times. The lysate was then centrifuged at 4500 rpm for 5 min at 4 °C. The pellet was washed twice by 1 ml cytoplasmic lysis buffer and centrifuged at 4500 rpm for 5 min at 4 °C. The nuclear pellet was then lysed by adding an appropriate volume of nuclear lysis buffer (20 mM Hepes [pH 7.9], 400 mM NaCl, 1 mM EDTA, 1 mM EGTA, and protease inhibitors [1:100]) and incubated on ice for 1 h. Finally, the mixture was centrifuged at 14,000 rpm for 10 min at 4 °C, and the supernatant, containing nuclear proteins, was collected.

### Western blotting analysis

Cultured cells were washed with PBS for three times. The cell pellet was lysed in total protein lysis buffer [50 mM Tris-HCl (pH 7.5), 150 mM NaCl, 1% Triton X-100, and protease inhibitor]. Protein concentration was determined by BCA assay (Cat.no. P0009, Beyotime Biotechnology). Equal amounts of protein samples were mixed with an appropriate volume of sample buffer and boiled at 100 °C for 10 min. Proteins were separated by SDS-PAGE and subsequently transferred to a nitrocellulose membrane. The membrane was blocked with 5% skim milk at RT for 1 h, followed by incubating with primary antibodies at 4 °C overnight. Primary antibodies were diluted 1:1000 and applied as follows: c-Myc (SC-40, Santa Cruz), actin (TA-09, ZSGB-BIO), DRP1 (611,113, BD Biosciences), MFN1 (13798-1-AP, Proteintech), MFN2 (12186-1-AP, Proteintech), ENO3 (15421-1-AP, Proteintech), Matrin3 (12202-2-AP, Proteintech), TFAM (ab131607, Abcam), OPA1 (27733-1-AP, Proteintech), mtCO2 (55070-1-AP, Proteintech), PGC1-α (NBP104676, Novus), cleaved PARP (9541S, CST), ATPB (17247-1-AP, Proteintech), RNaseH1 (15606-1-AP, Proteintech), Poly-γ (A8451, Abclonal), VDAC (55259-1-AP, Proteintech), Cytochrome-C (10993-1-AP, Proteintech), and MnSOD (611580, BD Biosciences). Next, the membranes were washed with tris-buffered saline containing 0.1% Tween-20 for three times and then incubated with horseradish peroxidase–conjugated secondary antibodies for 1 h at RT. The immunoreactive proteins were then visualized in chemiluminescence image analysis system. The bands were quantified to get grayscale values by ImageJ software.

### Quantitative real-time PCR

Cells were collected 72 h post-transfection, and genomic DNA were extracted using the Genomic DNA Extraction Kit (DP304–03, TIANGEN). The transcriptional levels of mtDNA within the genome were analyzed. qPCR was performed using the AceQ qPCR SYBR Green Master Mix (Q111-02, Vazyme) on an Applied Biosystems QuantStudio 3 system (Thermo Fisher Scientific). The list of primers used is as follows: m-nucGAPDH-F: 5′-GGACCTCATGGCCTACATGG-3′; m-nucGAPDH-R: 5′-AGGGCCTCTCTTGCTCAG-3′; m-mtDloop-F: 5′-CCCTTCCCCATTTGGTCT-3′; m-mtDloop-R: 5′-TGGTTTCACGGAGGATGG-3′; m-mtCO1-F: 5′-CTGAGCGGGAATAGTGGGTA-3′; m-mtCO1-R: 5′-GGGGCTCCGATTATTAGTG-3′; m-mtCO2-F: 5′-TAGGGCACCAATGATACTGAAG-3′; m-mtCO2-R: 5′-CTTCTAGCAGTCGTAGTTCACC-3′; m-mtND2-F: 5′-AACCCACGATCAACTGAAGC-3′; m-mtND2-R: 5′-TTGAGGCTGTTGCTTGTGTG-3′.

### Cell staining and flow cytometry

Cells were washed three times with PBS and subsequently dissociated to generate a single-cell suspension, which was transferred to a 1.5 ml microcentrifuge tube. The cells were then incubated in the dark at 37 °C for 30 min with either 5 μM MitoSOX Red (M36008, Thermo Fisher Scientific) or 0.25 μM tetramethylrhodamine (T668, Thermo Fisher Scientific). Following incubation, cells were centrifuged at 3000 rpm for 5 min at RT, washed three times with PBS, and resuspended in 1 ml PBS. The prepared cell suspensions were then subjected to flow cytometric analysis using a BD FACSCalibur flow cytometer.

### Immunofluorescence

For mouse striatum sections, the mouse was anesthetized and then perfused with PBS and 4% paraformaldehyde to remove blood and fix tissues. Sections (40 μm) of fixed tissues were prepared in cryostat microtome (Leica). For cellular immunofluorescence, the indicated cells were fixed in 4% paraformaldehyde for 20 min at 4 °C. The sections or fixed cells were then permeabilized with 0.2% Triton X-100 for 10 min at RT and blocked with 5% donkey serum containing 0.1% Triton X-100 for 1 h at RT, followed by incubating with primary antibodies diluting in 0.1% TritonX-100 and 5% donkey serum overnight at 4 °C. Next, the sections and cells were washed with PBS for three times and incubated with second antibodies for 1h. The coverslips were then mounted after washing with PBS for three times. Images were visualized and captured by a TS100 microscope (Nikon) or an N-SIM Structured Illumination Microscope (Nikon). The average length of mitochondrial branches was analyzed by ImageJ (Fiji). Briefly, the images were imported into ImageJ for preprocessing, which included unsharp masking, contrast enhancement, followed by median filtering to reduce noise. The images were then binarized and skeletonized to be analyzed by plugin (Analyze Skeleton). The total number of branches and the length of each branch are subsequently used for statistical analysis.

### Statistical analysis

All the data have passed normality test by Shapiro–Wilk test. Statistical analysis was performed using GraphPad Prism 8 software. Data are presented as the mean ± S.E.M. from three independent experiments. Unpaired *t* test was conducted for the comparison of two groups of samples. One-way ANOVA was applied for the statistical comparison between more than two groups, followed by Tukey's multiple comparison test or Dunnett's multiple comparisons test. Statistical significance was defined as *p* < 0.05 (∗), with higher levels of significance indicated by *p* < 0.01 (∗∗), *p* < 0.001 (∗∗∗), and *p* < 0.0001 (∗∗∗∗).

## Data availability

No data were used for the research described in the article.

## Supporting information

This article contains supporting information.

## Conflict of interest

The authors declare that they have no conflicts of interest with the contents of this article.

## Figures and Tables

**Fig. S1:**