# Supplementary material for: Leucine 7 is a key residue for mutant huntingtin–induced mitochondrial pathology and neurotoxicity in Huntington's disease
Source: J Biol Chem. 2025 Feb 11;301(3):108297. doi: 10.1016/j.jbc.2025.108297 (PMC11930128; doi:10.1016/j.jbc.2025.108297)
Supplement: Supporting Information [file mmc1.docx]

**Supporting Information Text**

**Fig.S1 The mutation of leucine 7 to alanine inhibits the mitochondrial association of HTT-Q73.**

**A-C.** Western blotting and quantitative analysis of the expression of Myc tagged HTT-Q23, HTT-Q73, HTT-Q73-L7A, HTT-Q73-L7I and HTT-Q73-L7D in WCL and mitochondrial fractions of SH-SY5Y cell at 72 h post transfection. n = 3 independent experiments. VDAC: loading control of mitochondrial fractions. One-way ANOVA was conducted in the above results, followed by Tukey’s multiple comparison test. **P* < 0.05.
